# Predictive factors for post-embolization infarction and long-term splenic remodeling in patients undergoing splenic artery aneurysm embolization

**DOI:** 10.3389/fsurg.2026.1737663

**Published:** 2026-05-07

**Authors:** Chang Hoon Oh, Kyoung Yeon Lee, Sang Yub Lee, Kwang Bo Park, Dongho Hyun, Yang-Jin Park, Shin-Seok Yang, Joon-Kee Park

**Affiliations:** 1 Department of Radiology, Samsung Medical Center, Sungkyunkwan University School of Medicine, Seoul, Republic of Korea; 2 Department of Radiology, CHA Bundang Medical Center, CHA University School of Medicine, Seongnam, Gyeonggi-do, Republic of Korea; 3 Division of Vascular Surgery, Department of Surgery, Samsung Medical Center, Sungkyunkwan University School of Medicine, Seoul, Republic of Korea

**Keywords:** embolization, endovascular therapy, splenic artery aneurysm, splenic infarction, splenic volume

## Abstract

**Objective:**

To identify predictive factors for post-embolization splenic infarction and to assess long-term remodeling patterns of the spleen, including regeneration and atrophy, in patients undergoing embolization for splenic artery aneurysm (SAA).

**Methods:**

This retrospective study included 64 patients (66 cases) with splenic artery aneurysms who underwent transcatheter arterial embolization between February 2007 and November 2023 at a single tertiary center. Embolization techniques included sac packing, trapping, combined approaches, and stent-graft placement.

**Results:**

Technical success was achieved in all 66 cases. Clinical success was obtained in 94.1% of evaluable patients, with two cases of recanalization and one new aneurysm detected during follow-up. Splenic infarction occurred in 24 patients (37.5%) and was associated with distal or hilar/intrasplenic location (*p* = 0.007), larger pre-embolization splenic volume (*p* = 0.018), and reduced splenic staining on angiography after embolization (*p* < 0.001). In multivariate analysis, only reduced splenic staining on angiography remained an independent predictor of infarction (HR = 0.939; *p* = 0.001). Among infarcted patients, 12 (50%) demonstrated regeneration and 12 (50%) progressed to atrophy, with regeneration associated with higher post-embolization splenic staining on angiography (*p* = 0.036), smaller infarcted volume (*p* = 0.003), and greater final splenic volume (*p* = 0.018). Overall complication rate was 39.1%, all of which resolved with conservative management. No major complications occurred.

**Conclusion:**

Endovascular embolization for splenic artery aneurysms was safe and effective. Post-embolization splenic staining on angiography was the key predictor of infarction and was also associated with long-term splenic regeneration.

## 1 Introduction

Splenic artery aneurysm (SAA) is a rare but clinically significant vascular disease, with an estimated prevalence of 0.09%–0.8% in the general population (1, 2). The major clinical concern with SAAs is the risk of rupture, which occurs in up to 10% of cases and is associated with mortality rates of 10%–25% in nonpregnant patients and up to 70% during pregnancy (3–6).

Endovascular therapy has become the preferred treatment owing to its minimally invasive nature, high technical success, and favorable outcomes compared with surgery (7). Endovascular embolization strategies include sac packing, trapping, combined techniques, and stent-graft placement (8, 9). Sac packing fills the aneurysm sac with coils while preserving parent artery patency. It is preferred for narrow-necked saccular aneurysms to maintain perfusion but carries a risk of recanalization if packing is incomplete (8, 10). Trapping involves occluding the parent artery proximal and distal to the aneurysm. It is typically used for fusiform or wide-necked aneurysms where sac packing is not feasible; while it ensures complete exclusion, it sacrifices parent artery flow and increases the risk of splenic infarction (8, 11). The combined technique employs both methods to reinforce occlusion in complex cases with backflow concerns. Finally, stent-graft placement excludes the aneurysm while preserving splenic perfusion, but its application is limited to vessels with favorable anatomy and minimal tortuosity (8).

Splenic infarction is one of the most common complications after embolization, and its extent has been closely associated with clinical outcomes. Smaller infarction volumes (≤50%) may allow preservation or even regeneration of splenic function, whereas extensive infarction has been linked to increased post-procedural morbidity (12–19). Follow-up studies further indicate that residual splenic tissue can regenerate after embolization, while chronic infarcted areas frequently progress to atrophy with compensatory hypertrophy of the remaining parenchyma (12–16). Nevertheless, the predictors of infarction and the long-term oucomes of splenic remodeling remain insufficiently characterized.

The present study aimed to identify predictive factors for post-embolization splenic infarction and to assess long-term remodeling patterns of the spleen, including regeneration and atrophy, in patients undergoing embolization for SAAs.

## 2 Materials and methods

### 2.1 Patients

This retrospective, single-center study was approved by the institutional review board, which waived the need for obtaining informed consent from the patients (SMC 2025-10-023-001). Between February 2007 and November 2023, medical records and imaging data of consecutive patients with splenic artery aneurysms who underwent transcatheter arterial embolization (TAE) at our institution were retrospectively analyzed. A total of 64 patients (66 cases) were included in the analysis. The inclusion criteria were patients aged 40–90 years with either true or pseudoaneurysms confirmed on computed tomography (CT). Exclusion criteria were an expected life expectancy of less than three months, poor general health status (Eastern Cooperative Oncology Group performance status grade 4), incomplete imaging data, and loss to follow-up.

### 2.2 Treatment techniques

The right common femoral artery was accessed under local anesthesia, and a 5-F RH catheter (Cook, Bloomington, IN, USA) was advanced with a 0.035-inch hydrophilic guidewire (Radifocus; Terumo, Tokyo, Japan) for selective catheterization of the celiac trunk, followed by celiac angiography. When further delineation of splenic artery branches arising from the aneurysm was required, the 5-F catheter was positioned in the celiac trunk or proximal splenic artery, and DynaCT imaging was performed to confirm the vascular anatomy. Based on angiographic and/or DynaCT findings, superselective catheterization of the aneurysm was achieved using a 1.7–2.0 Fr coaxial microcatheter (Progreat; Terumo, Tokyo, Japan, or Parkway; ASHAI INTECC, Aichi, Japan). The choice among the various techniques, including sac packing, trapping, combined sac packing and trapping technique, and stent-graft placement, was determined by the aneurysm's anatomical characteristics and operator preference.

**Figure 1:**
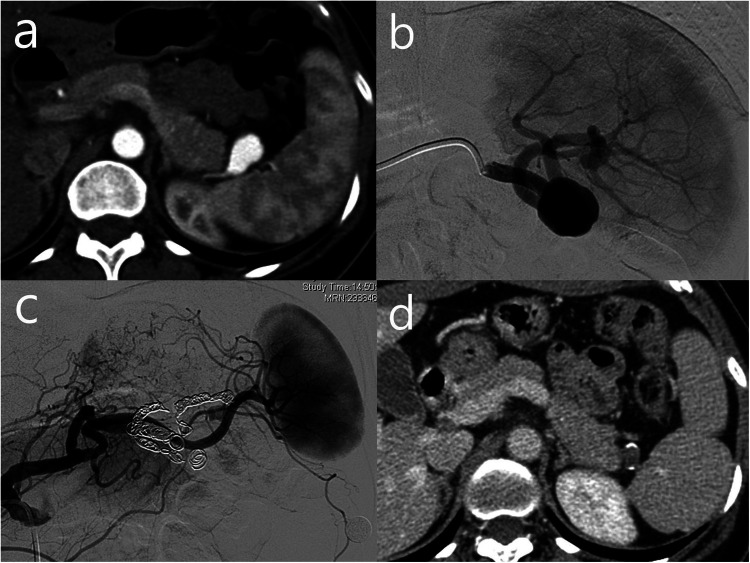
A 51-year-old female with a true splenic artery aneurysm located in the distal splenic artery, treated with the trapping technique. **(a)** Initial CT image shows a saccular aneurysm measuring 23.4 mm in maximum diameter, and the pre-treatment splenic volume was 194 mL. **(b)** Angiography demonstrates the aneurysm in the distal splenic artery with two distal branches arising from the aneurysmal sac. **(c)** Both the proximal and distal branches of the aneurysm were selected and embolized with a total of 11 coils. Final angiography confirmed complete exclusion of the aneurysmal flow with nearly preserved splenic parenchymal staining. **(d)** Follow-up CT obtained five years after embolization shows complete thrombosis with peripheral calcification and a decrease in the aneurysm size to 13 mm, without definite evidence of splenic infarction.

The trapping technique was the most frequently employed method, particularly in cases of pseudoaneurysms. It was also mainly used for large true SAAs, in which both the proximal and distal segments of the parent artery were embolized, with or without coil insertion into the aneurysm sac. Successful trapping was defined as complete exclusion of the aneurysm sac without residual opacification or distal runoff on final angiography (Figure 1).

The sac packing technique was primarily applied to true SAAs with narrow necks. In this approach, the aneurysm sac was tightly filled with detachable coils (Interlock; Boston Scientific, Marlborough, MA, USA, or Concerto; Medtronic, Minneapolis, MN, USA). Coils were chosen to match the sac diameter to achieve stable occlusion while reducing the risk of rupture or coil displacement. The combined technique involved both sac packing and trapping. In practice, distal branches were embolized with microcoils for trapping, and when backflow or potential recanalization was a concern, additional sac packing was performed to achieve complete exclusion of the aneurysm.

Stent-graft placement was considered for aneurysms located in the splenic artery trunk with sufficient proximal and distal landing zones and relatively straight vessel anatomy. After localization by angiography, a stent-graft (Viabahn; W.L. Gore & Associates, Flagstaff, AZ, USA, or Covera Plus; Bard, Tempe, AZ, USA) was deployed, typically 10% oversized relative to the parent artery and extending at least 1 cm beyond the aneurysm neck on both sides.

### 2.3 Follow up

After endovascular treatment for SAAs, periodic surveillance with contrast-enhanced CT is recommended to evaluate for endoleak or aneurysm reperfusion, which may result in aneurysm growth or rupture. Follow-up imaging was performed before discharge, at 1 month, at 6 and 12 months, and annually thereafter. In cases where patients developed abdominal pain, fever, or other abnormal symptoms, additional CT evaluations were performed as clinically indicated.

### 2.4 Study endpoints

Technical success was defined as complete aneurysm exclusion at ultimate angiographic control. Clinical success was defined as the absence of aneurysm reperfusion, recanalization, or the development of new aneurysms during follow-up, excluding cases in which assessment was limited by beam-hardening artifacts. Splenic staining after embolization on angiography was defined as the proportion (%) of the spleen showing parenchymal opacification on completion angiography after embolization, relative to the extent of splenic staining observed on pre-procedural angiography, excluding areas of perfusion defect. Complications were classified into minor and major categories according to Society of Interventional Radiology criteria (20). Splenic infarction was defined on contrast-enhanced CT as a peripheral, wedge-shaped hypodense lesion with its base at the splenic capsule and apex directed toward the hilum (21). After splenic infarction, regeneration was defined as an increase in splenic volume during follow-up, whereas atrophy was defined as a further decrease in splenic volume, based on serial CT volumetric measurements.

### 2.5 Evaluation of splenic volume and infarction

Splenic volume was quantitatively measured at three time points—pre-embolization, immediate post-embolization, and the final follow-up—using automated volumetric analysis software (Aquarius, TeraRecon, Inc.). Splenic infarction was diagnosed radiologically based on hypoenhancing areas on post-treatment CT scans. And splenic volume changes were quantitatively assessed, and post-procedural outcomes were categorized as atrophy or regeneration.

### 2.6 Statistical analysis

Categorical variables were compared using the chi-square test or Fisher's exact test, as appropriate. Continuous variables were expressed as the mean ± standard deviation and were compared using Student's t-test. Multivariate analysis was performed to identify independent factors using binomial logistic regression. All statistical analyses were performed using the SPSS software version 25.0, with a *p* value of <0.05 indicating statistical significance.

## 3 Results

Baseline demographic and clinical characteristics are summarized in Table 1. True aneurysms accounted for 90.9% (*n* = 60), while pseudoaneurysms were identified in 9.1% (*n* = 6). The mean follow-up duration for the entire cohort (*n* = 66) was 1,237.5 ± 1,322.5 days. The majority of aneurysms were located in the distal (*n* = 24, 36.4%) or hilar/intrasplenic regions (*n* = 17, 25.7%), followed by mid (*n* = 15, 22.7%) and proximal (*n* = 10, 15.2%) segments. The mean aneurysm diameter was 27.2 ± 12.1 mm, and the mean pre-embolization splenic volume was 377.4 ± 346.4 cm^3^. Embolic materials consisted primarily of only microcoils (74.2%), followed by combined microcoil and vascular plug (12.1%), vascular plug alone (4.5%), microcoil with vascular plug and NBCA (4.5%), and stent-graft (4.5%). Trapping was the most frequently used embolization technique (71.2%), while sac packing (7.6%) and combined sac packing with trapping (19.7%) were less common. Of the 66 cases, 32 (48.5%) had no underlying disease. Hypertension and atherosclerosis were present in 24.2% and 13.6% of cases, respectively. Other cardiovascular diseases, diabetes mellitus, and pancreatitis were less common.

**Table 1:** Baseline demographics and clinical data of the study patients.

Characteristics	Patients (*n* = 64), Treated lesion (*n* = 66)
Sex (%)	
Male	33 (51.6)
Female	31 (48.4)
Mean age, years (range)	62.09 ± 12.69
Underlying disease (%)	
Hypertension	16 (24.2)
Atherosclerosis	9 (13.6)
Other cardiavascular disease*	4 (6.1)
DM	2 (3.0)
Pancreatitis	1 (1.5)
Hospital stay (days)	7.32 ± 11.00
Aneurysm (%)	
True aneurysm	60 (90.9)
Pseudoaneurysm	6 (9.1)
Aneurysm location (%)	
Proximal	10 (15.2)
Mid	15 (22.7)
Distal	24 (36.4)
Hilum	15 (22.7)
Splenic parenchyma	2 (3.0)
Embolic materials (%)	
Only microcoil	49 (74.2)
Only vascular plugs	3 (4.5)
Microcoil ± Vascular Plug	8 (12.1)
Microcoil ± Vascular Plug ± NBCA	3 (4.5)
Only Stent-graft	1 (1.5)
Stent-graft ± microcoil	2 (3.0)
Embolization technique (%)	
Only sac packing	5 (7.6)
Only trapping	47 (71.2)
Combination of sac packing and trapping	13 (19.7)
Only stent-graft	1 (1.5)
Splenic volume (before embolization, cm^3^)	377.43 ± 346.40
Aneurysm diameter (maximal diameter, mm)	27.20 ± 12.13

* Includes ischemic heart disease (*n* = 2), abdominal aorta aneurysm (*n* = 1), and superior mesenteric artery aneurysm (*n* = 1).

Technical success was achieved in all patients. After excluding 15 cases with nondiagnostic follow-up imaging due to beam-hardening artifacts, clinical success was observed in 48 of 51 evaluable cases (94.1%). Recanalization occurred in two patients, both of whom had been treated with the trapping technique without sac packing; in these cases, contrast opacification of the aneurysm cavity was observed. However, in both patients with recanalization, the aneurysm size remained stable without further enlargement over prolonged follow-up periods (4,310 and 1,547 days, respectively). As both patients remained asymptomatic and the aneurysms showed no signs of growth, they were managed with expectant management and close imaging surveillance without secondary intervention. One patient developed a new aneurysm in a more proximal segment of the splenic artery during follow-up.

Table 2 shows patient characteristics stratified by the occurrence of splenic infarction. Splenic infarction occurred in 24 patients (37.5%). Aneurysm location differed significantly between the groups (*p* = 0.007). Infarction occurred more frequently in distal or hilar/intrasplenic aneurysms, while proximal aneurysms were observed only in the non-infarction group. Patients with infarction had larger mean splenic volume before embolization compared with those without infarction (509.4 ± 454.5 cm^3^ vs. 302.0 ± 241.6 cm^3^, *p* = 0.018). The extent of embolization was also associated with infarction (*p* < 0.001), with hilar/intrasplenic embolization showing the highest proportion. Splenic staining after embolization on angiography was significantly lower in patients with infarction compared with those without (50.4 ± 17.6% vs. 81.0 ± 22.3%, *p* < 0.001). Aneurysm type, aneurysm size, and embolization technique did not show statistically significant differences between the groups.

**Table 2:** Patient characteristics according to the presence or absence of splenic infarction.

Characteristics	Splenic infarction		*P* value
	Yes (*n* = 24)	No (*n* = 42)	
Sex			0.709
Male	12	23	
Female	12	19	
Age	62.79 +/− 10.06	61.69 +/− 14.07	0.714
Aneurysm location			0.007
Proximal	0	10	
Mid	4	11	
Distal	9	15	
Hilum∼intrasplenic parenchyma	11	6	
Aneurysm			0.106
True aneurysm	20	40	
Pseudoaneurysm	4	2	
Aneurysm size	27.76 +/− 10.87	26.89 +/− 12.91	0.771
Volume (before embolization, cm^3^)	509.42 +/− 454.46	302.02 +/− 241.57	0.018
Embolic extent			<0.001
Proximal	0	6	
Mid	2	9	
Distal	4	20	
Hilum∼intrasplenic parenchyma	18	7	
Embolic technique			0.069
Sac packing	0	5	
Trapping	16	31	
Combination (Sac packing + Trapping)	8	5	
Only stent-graft	0	1	
Splenic staining after embolization on angiography (%)	50.4 +/− 17.6	81.0 +/− 22.3	<0.001

Univariate logistic regression identified larger pre-embolization splenic volume (HR = 1.002; 95% CI, 1.000–1.003; *p* = 0.031), embolic extent distal to the intrasplenic parenchyma (HR = 6.111; 95% CI, 1.260–29.644; *p* = 0.025), and splenic staining after embolization on angiography (HR = 0.934; 95% CI, 0.902–0.967; *p* < 0.001) as significant risk factors for infarction. In the multivariate model, only splenic staining after embolization on angiography remained statistically significant (HR = 0.939; 95% CI, 0.904–0.975; *p* = 0.001) (Table 3).

**Table 3:** Univariate and multivariate logistic regression analysis of risk factors associated with splenic infarction.

Variable	Univariate analysis			Multivariate analysis		
	HR	95% CI	*P*	HR	95% CI	*P*
Sex						
Male	0.632	0.302–2.256	0.213			
Female						
Age	0.371	0.968–1.048	0.371			
Aneurysm						
True aneurysm	0.250	0.042–1.483	0.127			
Pseudoaneurysm						
Aneurysm location						
Distal to intrasplenic parenchyma	2.584	0.808–8.259	0.109			
Proximal to mid						
Volume (before embolization,)	1.002	1.000–1.003	0.031	1.000	0.998–1.002	0.748
Aneurysm size	1.006	0.965–1.048	0.777			
Embolic extent						
Distal to intrasplenic parenchyma	6.111	1.260–29.644	0.025	3.611	0.491–26.531	0.207
Proximal to mid						
Angiographic splenic staining after embolization (%)	0.934	0.902–0.967	<0.001	0.939	0.904–0.975	0.001

**Figure 2:**
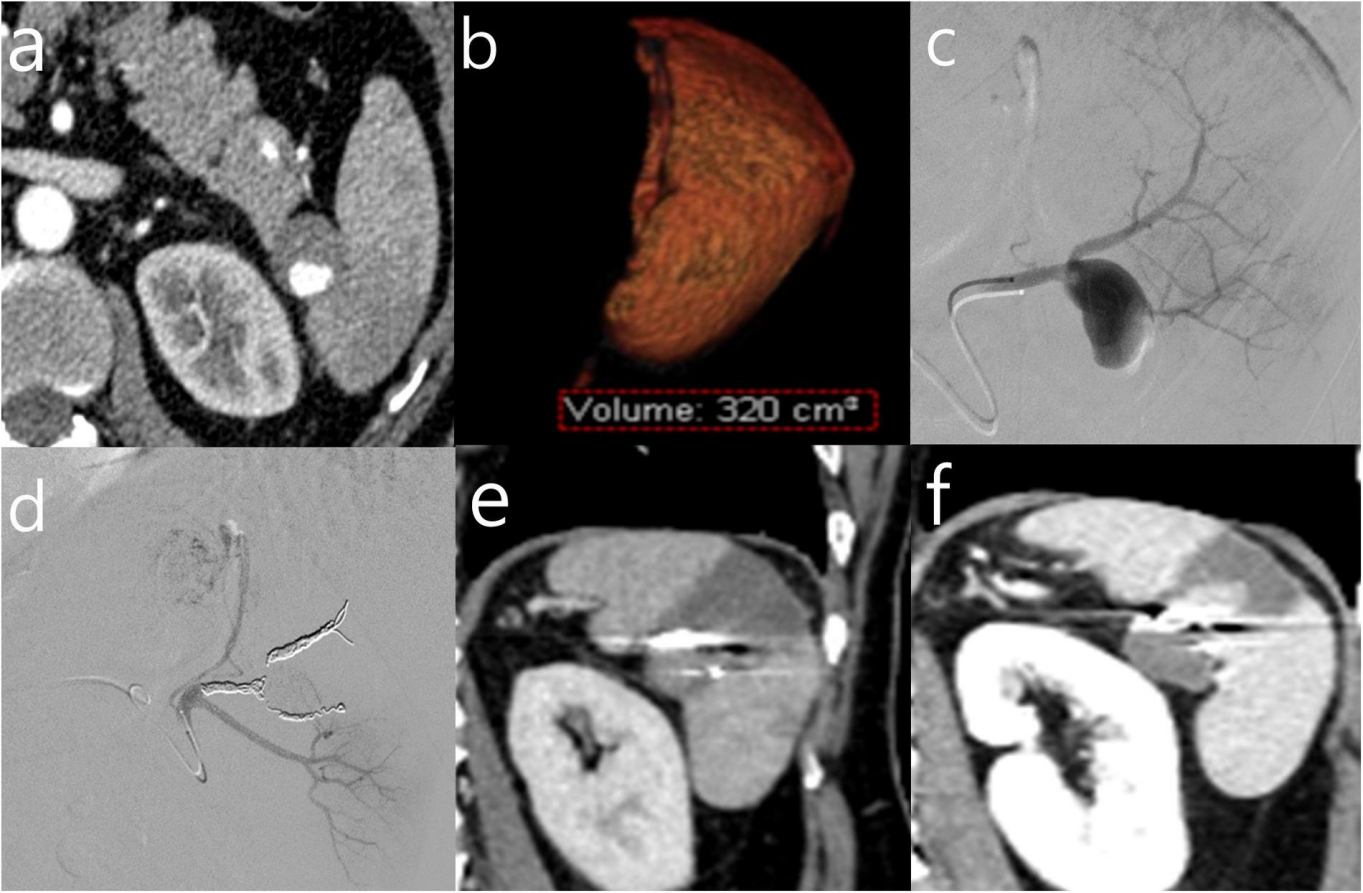
Panel a shows a CT scan revealing a saccular aneurysm at the splenic hilum measuring 23.3 mm. Panel b displays a 3D volumetric rendering of the spleen with a labeled volume of 320 cm³. Panel c shows an angiographic image with the hilar aneurysm and two distal branches. Panel d shows selective embolization of the proximal and distal branches with microcoils. Panel e is a follow-up CT seven days post-embolization showing splenic infarction involving approximately 62.5% of the spleen with a volume of 218 mL. Panel f is a follow-up CT at 25 days showing partial splenic regeneration with an increased volume of 243 mL

Among the 24 patients who developed splenic infarction, splenic regeneration at follow-up was observed in 12 patients (50.0%), whereas the remaining 12 patients (50.0%) showed progressive atrophy. Post-embolization symptoms occurred in 16 patients (66.7%), while 8 patients (33.3%) remained asymptomatic. The mean pre-embolization splenic volume was 482.0 ± 432.5 cm^3^, which decreased to 228.5 ± 213.5 cm^3^ after embolization. The mean infarction ratio was 50.8% ± 22.4%, and the last follow-up splenic volume was 245.9 ± 199.1 cm^3^. Table 4 summarizes patient characteristics according to regeneration or atrophy after splenic infarction. Patients in the regeneration group demonstrated higher splenic staining after embolization on angiography (54.2% ± 18.8 vs. 40.0% ± 10.4, *p* = 0.036). The proportion of infarcted splenic volume was significantly lower in the regeneration group compared with the atrophy group (38.1% ± 20.1 vs. 63.5% ± 16.3, *p* = 0.003). In addition, the last follow-up splenic volume was significantly greater in patients with regeneration than in those with atrophy (339.7 ± 152.2 cm^3^ vs. 152.1 ± 201.3 cm^3^, *p* = 0.018) (Figure 2).

**Table 4:** Patient characteristics according to the regeneration or atrophy after splenic infarction.

Characteristics	Regeneration (*n* = 12)	Atrophy (*n* = 12)	*P* value
Sex (%)			0.102
Male	8 (66.7)	4 (33.3)	
Female	4 (33.3)	8 (66.7)	
Age	62.75 +/− 11.23	62.83 +/− 9.24	0.984
Embolization extent (%)			0.143
Proximal	0 (0)	0 (0)	
Mid	2 (16.7)	0 (0)	
Distal	3 (25.0)	1 (8.3)	
Hilum∼intrasplenic	7 (58.3)	11 (91.7)	
Abnormal symptoms after embolization (%)			0.386
Yes	7 (58.3)	9 (75.0)	
No	5 (41.7)	3 (25.0)	
Duration of post embolization syndrome (days)	2.83 +/− 3.13	4.50 +/− 4.28	0.289
Splenic staining after embolization on angiography (%)	54.2 +/− 18.8	40.0 +/− 10.4	0.036
Volume (before embolization, cm^3^)	467.92 +/− 433.19	496.00 +/− 450.52	0.878
Infarction volume (cm^3^)	274.00 +/− 362.31	287.75 +/− 248.79	0.915
% of Infarcted area volume	38.1 +/− 20.1	63.5 +/− 16.3	0.003
Volume (last follow-up, cm^3^)	339.67 +/− 152.20	152.08 +/− 201.30	0.018

A total of 25 patients (39.1%) experienced post-procedural complications, including 24 with splenic infarction and 1 with acute pancreatitis. Among them, 16 patients were considered to have post-embolization syndrome, presenting with fever, left flank pain, or inflammatory marker elevation. Patients with post-procedural complications had a longer mean hospital stay compared to those without (11.3 ± 16.7 days vs. 4.9 ± 3.7 days; *p* = 0.068), reflecting the need for in-hospital symptom management. The mean duration of post-embolization syndrome was 3.7 ± 3.8 days (range, 1–15 days). All complications were successfully managed with conservative treatment, which consisted of supportive care including analgesia, antipyretics, and intravenous hydration, and symptoms resolved without further intervention. No major complications occurred, and none of the patients with splenic infarction developed secondary infection or other adverse sequelae.

## 4 Discussion

In our study, technical success was achieved in all cases, with a high clinical success rate (94.1%) and no major complications. Splenic infarction (37.5%) was the most frequent complication; although these patients showed a trend toward longer hospitalization for supportive care (11.3 ± 16.7 vs. 4.9 ± 3.7 days; *p* = 0.068), this difference was not statistically significant, and all cases resolved with conservative management alone without additional intervention. These findings are consistent with previous studies reporting that splenic infarction is relatively common after embolization but is usually self-limiting and rarely associated with adverse sequelae (11, 13, 18, 19). Prior studies have also reported high efficacy of endovascular embolization, with documenting technical success rates exceeding 95% with preserved splenic perfusion in most patients (2), and a 98% success rate across various SAA types with durable long-term exclusion (22). Furthermore, even when the main splenic artery is occluded, substantial residual perfusion may be maintained via collateral circulation (23, 24), a finding that is in line with our observation of splenic regeneration and stable outcomes in many cases.

In our study, on logistic regression analysis, aneurysm location was not a significant predictor (HR = 2.584; *p* = 0.109), whereas embolization extent distal to the intrasplenic parenchyma emerged as a significant factor (HR = 6.111; *p* = 0.025). These findings indicate that the risk of infarction depends less on the anatomical location of the aneurysm itself and more on how far the embolization extends into the distal splenic artery branches. Trapping, the most frequently used technique in our study, inherently requires occlusion of distal splenic arterial branches and has consistently been associated with higher rates of infarction in previous studies (8, 11). In line with this, Devos et al. demonstrated that distal embolization led to an average 30% reduction in splenic volume (*p* = 0.003), and that marked volume loss (>50%) was strongly correlated with coil extension into one or more hilar branches (*p* = 0.005) (23). These findings reinforce the concept that embolization extending into distal or hilar branches is a critical determinant of long-term splenic volume loss and the risk of clinically significant infarction.

In our study, univariate analysis showed that splenic infarction was associated with pre-embolization splenic volume, embolization extent, and splenic staining after embolization on angiography. Among these, only splenic staining remained significant in multivariate analysis (HR = 0.939, *p* = 0.001). Notably, even among patients with infarction, those achieving regeneration retained higher residual staining (54.2 ± 18.8%) compared to those progressing to atrophy (40.0 ± 10.4%). Collectively, these findings suggest that a residual staining of less than 50% serves as a critical angiographic threshold indicating a significantly increased risk of irreversible infarction and subsequent atrophy. While prior studies of partial splenic embolization have focused on embolized volume as a determinant of complications (13, 18, 19), our results underscore the value of post-embolization staining as an immediate, readily available marker of splenic viability.

In our study, among the 24 patients (37.5%) who developed splenic infarction, 12 (50.0%) demonstrated splenic regeneration during follow-up, whereas the remaining 12 (50.0%) progressed to atrophy. Regeneration was significantly associated with higher post-embolization parenchymal staining on angiography (*p* = 0.036), smaller infarcted volume (*p* = 0.003), and greater final splenic volume (*p* = 0.018), underscoring the importance of preserving sufficient perfusion and maintaining a relatively larger volume of viable parenchyma after embolization. Experimental studies in animal models have shown that the spleen is capable of regeneration, though not necessarily to its original volume, with preservation of architecture and lymphoid follicle hyperplasia within one month (25). Clinical investigations of partial splenic embolization have similarly demonstrated gradual increases in splenic volume beginning at six months, consistent with regeneration of residual tissue (12–15). In Zhu et al, 15 patients with splenic infarction ranging from 45%–81%, those with moderate infarction (45%–68%) exhibited progressive volume recovery and declining infarction rates over two years, whereas patients with more extensive infarction (≥70%) showed only minimal recovery, with infarction rates persisting around 70% (13). Collectively, these findings emphasize that balanced embolization, sufficient to exclude the aneurysm while preserving adequate splenic perfusion, is crucial for favorable long-term splenic remodeling.

Finally, regarding generalizability, the trapping technique was frequently employed in our cohort due to anatomical constraints, such as vascular tortuosity or distal aneurysm location, which often precluded the use of stent-grafts or sac packing. However, our findings are highly relevant to centers favoring these flow-preserving strategies. Since sac packing and stent-graft placement inherently maintain parent artery patency and preserve splenic perfusion, they are expected to yield high post-embolization angiographic staining. Our observation that high residual staining significantly reduces the risk of infarction therefore supports the rationale for using these flow-preserving techniques when anatomically feasible, while highlighting the critical importance of monitoring staining defects when trapping is unavoidable.

This study has several limitations. First, it was a retrospective analysis from a single tertiary center, which may have introduced selection bias and limits the generalizability of the findings. Second, the study population was heterogeneous in terms of underlying conditions, aneurysm morphology, embolic materials, and operator-dependent technique selection, which could have influenced outcomes. Third, follow-up evaluation relied exclusively on radiologic parameters, primarily CT volumetry and angiographic staining; functional assessment of splenic immunity was not performed. Moreover, in some cases, evaluation was limited by beam-hardening artifacts, which impeded accurate assessment of aneurysm reperfusion or remodeling. Although current guidelines recommend vaccination following splenectomy, our study population underwent partial embolization, and vaccination was not routinely performed or tracked. These observations point to several promising directions for future research. Prospective multicenter studies with larger cohorts are needed to validate the angiographic staining threshold identified here and to establish standardized criteria for predicting clinically significant infarction. Functional assessment of splenic immunity—such as measurement of immunoglobulin levels, opsonin activity, or Howell-Jolly body counts—should be incorporated into future follow-up protocols, as radiologic volumetry alone cannot capture the full immunological impact of post-embolization remodeling. The role of next-generation flow-diverting devices and liquid embolic agents in minimizing infarction while ensuring durable aneurysm exclusion also merits prospective evaluation. Finally, development of vaccination protocols tailored to patients with extensive post-embolization infarction remains an important unmet clinical need, given the theoretical risk of functional asplenia in this population. Finally, subgroup analyses of regeneration and atrophy were based on relatively small numbers, reducing statistical power and limiting the strength of the conclusions.

In conclusion, TAE for SAAs was safe and effective, with no major complications. Splenic parenchymal staining on angiography after embolization was the only independent predictor of infarction. Furthermore, splenic regeneration was more likely when post-embolization staining was higher, infarcted volume was smaller, and final splenic volume was greater.

## Data Availability

The raw data supporting the conclusions of this article will be made available by the authors, without undue reservation.
